# Biomass Production and Predicted Ethanol Yield Are Linked with Optimum Photosynthesis in *Phragmites karka* under Salinity and Drought Conditions

**DOI:** 10.3390/plants11131657

**Published:** 2022-06-23

**Authors:** Zainul Abideen, Hans Werner Koyro, Tabassum Hussain, Aysha Rasheed, Mona S. Alwahibi, Mohamed S. Elshikh, Muhammad Iftikhar Hussain, Faisal Zulfiqar, Simeen Mansoor, Zaheer Abbas

**Affiliations:** 1 Dr. Mouhammed Ajmal Khan Institute of Sustainable Halophyte Utilization, University of Karachi, Karachi 75270, Pakistan; thussain@uok.edu.pk (T.H.); halophyte_aysha@yahoo.com (A.R.); 2 Institute of Plant Ecology, Justus-Liebig-University Giessen, D-35392 Giessen, Germany; hans-werner.koyro@bot2.bio.uni-giessen.de; 3 Department of Botany and Microbiology, College of Science, King Saud University, Riyadh 11451, Saudi Arabia; malwhibi@ksu.edu.sa (M.S.A.); melshikh@ksu.edu.sa (M.S.E.); 4 Department of Plant Biology & Soil Science, Universidad de Vigo, Campus Lagoas Marcosende, 36310 Vigo, Spain; 5 Department of Horticultural Sciences, Faculty of Agriculture and Environment, Islamia University of Bahawalpur, Bahawalpur 63100, Pakistan; ch.faisal.zulfiqar@gmail.com; 6 Department of Genetics, University of Karachi, Karachi 75270, Pakistan; simeenm@uok.edu.pk; 7 Department of Botany, Division of Science and Technology, University of Education Lahore 54770, Pakistan; fzaheerbot@gmail.com

**Keywords:** bioethanol, salt tolerance, water deficit conditions, chlorophyll fluorescence, photosynthetic efficiency

## Abstract

Plant photosynthesis and biomass production are closely associated traits but critical to unfavorable environmental constraints such as salinity and drought. The relationships among stress tolerance, photosynthetic mechanisms, biomass and ethanol yield were assessed in *Phragmites karka*. The growth parameters, leaf gas exchange and chlorophyll fluorescence of *P. karka* were studied when irrigated with the control and 100 and 300 mM NaCl in a nutrient solution and water deficit conditions (drought, at 50% water holding capacity). The plant shoot fresh biomass was increased in the low NaCl concentration; however, it significantly declined in high salinity and drought. Interestingly the addition of low salinity increased the shoot biomass and ethanol yield. The number of tillers was increased at 100 mM NaCl in comparison to the control treatment. High salinity increased the photosynthetic performance, but there were no significant changes in drought-treated plants. The saturated irradiance (Is) for photosynthesis increased significantly in low salinity, but it declined (about 50%) in high salt-stressed and (about 20%) in drought-treated plants compared to the control. The rates of dark respiration (Rd) and compensation irradiance (Ic) were decreased significantly under all treatments of salinity and drought, with the exception of unchanged Rd values in the control and drought treatments. A-Ci curve analyses revealed a significant improvement in the Jmax, Vc, max, and triose-phosphate utilization (TPU) at lower salinity levels but decreased at 300 mM NaCl and drought treatments compared to the control. In the chlorophyll fluorescence parameters (Fv/Fm, maximum photochemical quantum yield of PSII, and Y(NO)), the non-photochemical yields were not affected under the salt and drought treatments, although an effective photochemical quantum yield (YII) and electron transport rate (ETR) were significantly enhanced in water deficit compared to control plants. *P. karka* regulates an efficient photosynthesis mechanism to grow in saline and arid areas and can therefore be used as a sustainable biofuel crop.

## 1. Introduction

Plants of arid and semi-arid regions display severely subdued growth and even death in the presence of either drought or saline conditions [1]. Species belonging to these conditions gradually exhibit lesser vegetation cover and can lead to desertification in the region [2]. NaCl stress and water deficit are common abiotic stress factors on a global scale and cause deleterious effects on plant biomass and stability [3,4,5]. Functionally, plants can reduce the harmful effects of water limitation and ion toxicity (due to soil salinity) by altering their growth, water relations, and photosynthesis [6,7,8]. Growth inhibition and leaf shedding under such conditions also help plants to maintain their water status and survive [9]. Most likely, biomass production in halophytes is related to photosynthesis and their protective photosystem (PS I and II) performances under salt stress [3,10]. Applying eco-physiological tools to assess the functional contribution of photosynthesis and their associated adjustments is important for biomass production [11,12,13]. 

The beneficial effects of low NaCl concentrations (100 mM) on growth and photosynthesis have been frequently observed in many studies [14,15]. It was reported that sodium ion acts as a cheap osmoticum for leaf turgor maintenance [14]. For instance, members of Chenopodiaceae attain benefits from sodium [16,17]. In C_4_ species, it was assumed that Na^+^ facilitate pyruvate conversion into phosphoenolpyruvate, found in mesophyll, before being added to the Calvin cycle. In addition, two halophytic species: *Kochia childsii* and *Atriplex tricolor* were cultivated in a sodium-deficient medium that declined photosystem II activities in mesophyll chloroplasts [18]. However, higher concentrations of Na^+^ have deleterious effects on the photosynthetic apparatus [15].

The declined carbon fixation in salt and drought-stressed plants is also linked to lower stomatal conductance, and therefore, disturbance in the flow of electrons to Photosystem II can be possible [13,14]. The deficiency of electron and proton acceptors causes excessive light to release a surplus amount of energy as heat and chlorophyll fluorescence in plants to prevent the production of reactive oxygen species (ROS) [19,20]. Stress-tolerant plants regulate the photosynthetic rate and photoprotective mechanism to reduce the deleterious effects of ROS, which are linked with the optimum ATP synthesis, and NADP formation [21,22]. The above-said parameters are very informative in assessing the photosynthesis and physiological performance of plants [11]. The understanding of carbon assimilation and energy conversion phenomenon are linked to the production of all types of bio-compounds (e.g., ethanol), and therefore, the full potential of plants can be utilized in stressed conditions. In particular, halophytes are much-suited candidates due to their natural distributions in extreme conditions (e.g., salinity and water deficit). 

It was recently demonstrated that *Phragmites karka* exhibits an efficient mechanism to tolerate salt and drought stresses, but a detailed analysis of their photochemistry and bioethanol potential is still poorly known [3]. In this study, *P. karka* plants coordinated changes involving the rate of photosynthesis and efficient photosystem II activity under saline and water deficit conditions. This plant accumulates a high amount of soluble sugar and lignocellulosic biomass [21,22]. This paper unlocks the potential of this accumulated sugar and cellulose and subsequent hemicellulose conversion into the ethanol yield on those areas that seem not suitable for agriculture. The effects of salt and drought stress on photosynthesis and their relationship with the biomass and ethanol yield was evaluated. The establishment of a suitable growing condition of the selected biofuel crop and subsequent ethanol potential can be helpful in the remediation of the increasing saline lands of Pakistan and other arid regions of the world. 

## 2. Material and Methods 

### 2.1. Plant Growth Conditions

Seeds of *Phragmites karka* were collected from the population located at the University of Karachi, Pakistan. A growth experiment was carried out under controlled growth chamber conditions in a growth chamber in Giessen, Germany: optimum temperature of 25 ± 2 °C, relative humidity around 50%, and photoperiod 16–8 h day–night, while the light intensity was 200–250 μmol m^−2^ s^−1^ photosynthetically active radiations. In the beginning, plant seeds were germinated in the plastic tray with wet clay soil for seedling emergence. Wuxal Super (Aglukon, Düsseldorf, Germany) was used as the nutrient for a further seven weeks. Subsequently, seedlings were transplanted into the soil (composed of 50% sand, 30% clay, and 20% gravels) in plastic pots (35 cm in height, 11 cm in diameter, and three plants per pot). The plants were irrigated periodically (12 h, from 8 am to 8 pm) with a basic nutrient solution (1/2 strength Hoagland modified after Epstein, 1972 [23]) in a quick check system [24]. The pots were divided into four groups (at the age of thirty-five days (35) after seed germination): control, at 100% water holding capacity (WHC), low and high salinity (100 and 300 mM NaCl in a nutrient solution with 100% WHC), and drought (reduced water supply at 50% WHC). Each pot contains three plants, while there are eight pots for each treatment. Plants of the control treatment were irrigated only with a nutrient solution, whereas the salt concentration was stepwise raised by adding 50 mM NaCl per day until the final concentration of desired NaCl was reached in the growth medium. In parallel to the salinity experiment, a drought treatment was started by gradually (5% per day) reducing the soil water saturation from 100 to 50%. The water holding capacity was measured as described by Veihmeyer and Hendrickson (1931) [25]. The treatments were designed based on the preliminary growth trials. The plants of all four treatments were maintained for a further five weeks. At the end of the experimental period, the soil water potential was −1.5 MPa in salinity and −0.5 MPa in drought. After 10 weeks, the plants were harvested for an eco-physiological analysis (total age) under these conditions. 

### 2.2. Plant Harvest and Growth Parameters

Before the plant harvest, nondestructive growth parameters such as the predawn leaf water potential and midday gas exchange of leaves were recorded. The fresh weight (FW) of leaves, stems, and roots was noted. Plants were harvested and dried at 80 °C for 24 h for calculating the dry weight (DW). The leaf relative water content (RWC) was calculated separately using the equation:

LRWC (%) = [(FW − DW)/(TW − DW)] × 100 where TW is turgid weight

A leaf is converted into a small disc and immersed for three hours to attain full turgidity at room temperature. Turgid small discs are taken out from the water and dried immediately with the help of tissue paper. The dried leaf discs are quickly weighed to determine the turgid weight (TW).

### 2.3. Soil Water Potential

The water potential in the soil was assessed using Wescor soil in a psychrometer (attached to a data logger) when the soil dried had an initial moisture concentration of 50% WHC; all this took about 4 weeks. Weight was assessed on a daily basis during this course of time. Soil samples were dried in the oven at the end of the experiment; the dry samples were weighed to determine the constant weight and dry weight. Water loss (up to 50% WHC for dry soil) was detected as a weight loss, which relates to the soil’s ability to hold water. This function is used to calculate potential water based on the known water content.

### 2.4. Leaf Gas Exchange and Chlorophyll Content 

LI-COR 6400 (LI-COR, Lincoln, NE, USA) was used to determine the gas exchange parameter, with 400 µmol m^−2^ s^−1^ CO_2_ and a 500 µmol m^−2^ s^−1^ flow rate. Different PAR values ranged 0–2000 μmol photon m^−2^ s^−1^ to calculate the dark respiration (Rd), compensation irradiance (Ic), saturation irradiance (Is), and photosynthetic efficiency (Φc), as described by [26]. In contrast, different CO_2_ concentrations were plotted to calculate the maximum Rubisco carboxylase activity (Vc, max) and maximum rate of electron transport to regenerate RuBP (Jmax) and triose-phosphate utilization (TPU) [27]. The relative chlorophyll content was measured using a SPAD 502 densitometer (Konica Minolta, Ramsey, NJ, USA).

### 2.5. Leaf Chlorophyll Fluorescence

The chlorophyll fluorescence was measured on similar leaf sections to those selected for the gas exchange (Pulse-controlled Junior PAM, Walz, Effeltrich, Germany). The leaves were kept in complete dark for 30 min to determine the following parameters, as described in Abideen et al. (2020) [28]: minimal fluorescence (Fo) with modulated light (<0.1 μmol photon m^−2^ s^−1^) and maximal fluorescence (Fm) with saturating pulse (10,000 μmol photons m^−2^ s^−1^ for 0.6 s) determined the maximum photochemical quantum yield of PSII.

Maximum photochemical quantum yield of photosystem II (Fv/Fm) = Fm − Fo/Fm) [29].

Effective photochemical quantum yield YII = Fm’ − Fs/Fm’ and NPQ = Fm/Fm’ − 1 [30]. NPQ = Fm/Fm’ − 1 [30].

Non-photochemical quenching Y (NO) = F/FmY (NPQ) = F/Fm’ − F/Fm [31]. 

The coefficient of photochemical quenching (qP) = Fm’ − Fs)/(Fm’ − Fo’) [32]. 

Electron transport rate ETR = PSII × PPFD × 0.5 × 0.84 [33]. 

### 2.6. Lignocellulosic Analysis and Soluble Sugar Content

The lignocellulosic content was analyzed in dry shoots by the neutral detergent fiber (NDF) determination. The acid detergent fiber (ADF) was determined by using the residue left from the NDF analysis. Hemicellulose was determined by subtracting the ADF from NDF [34]. The ADF and NDF-treated shoot biomass were then hydrolyzed with 72% H_2_SO_4_ to determine the cellulose levels. Dry plant leaves were brought to a powdered form and shaken for an hour at 100 °C with deionized water, and the filtrate was obtained to treat with Anthrone’s reagent to calculate the soluble sugar. The mixture was heated in a boiling water bath for 11 min, followed by cooling at room temperature. The optical density of green to dark green color was observed at 630 nm on a spectrophotometer (DU530 UV–Vis) [35].

### 2.7. Statistical Analysis and Calculation

Data (*n* ≥ 4) were analyzed by one-way analysis of variance (ANOVA, SPSS, ver. 11), and significant differences among means (*p* < 0.05) were determined by the Bonferroni test.

The conversion of dry matter per pots into tons of biomass/hectare was performed by calculating the plant yield per pot [36]. Firstly, the surface area of the pot in cm² is calculated. Then, the unknown yield per hectare (x) is calculated in relation to the area that is 10,000 m², as shown by Zhao et al. [36]. The theoretical yield of ethanol cellulose and total soluble sugar data per hectare levels was determined by the following equation:

Ethanol yield from soluble sugar (L ha^−1^) = total soluble sugar content (%) in dry matte (t ha^−1^) × 0.51 (conversion factor of ethanol from sugar) × 0.85 (process efficiency of ethanol from sugar) × 1000/0.79 (specific gravity of ethanol, g mL^−1^) [36].

The ethanol yield from cellulose and hemicellulose (L ha^−1^) = cellulose and hemicellulose content (% DW) in dry matter × dry biomass (t ha^−1^) × 1.11 (conversion factor of sugar from cellulose and hemicellulose) × 0.85 (process efficiency of sugar from cellulose and hemicellulose) × 0.51 (conversion factor of ethanol from sugar) × 0.85 (process efficiency of ethanol from sugar) × 1000/0.79 (specific gravity of ethanol, g mL^−1^) [36].

## 3. Results

**Figure 1:**
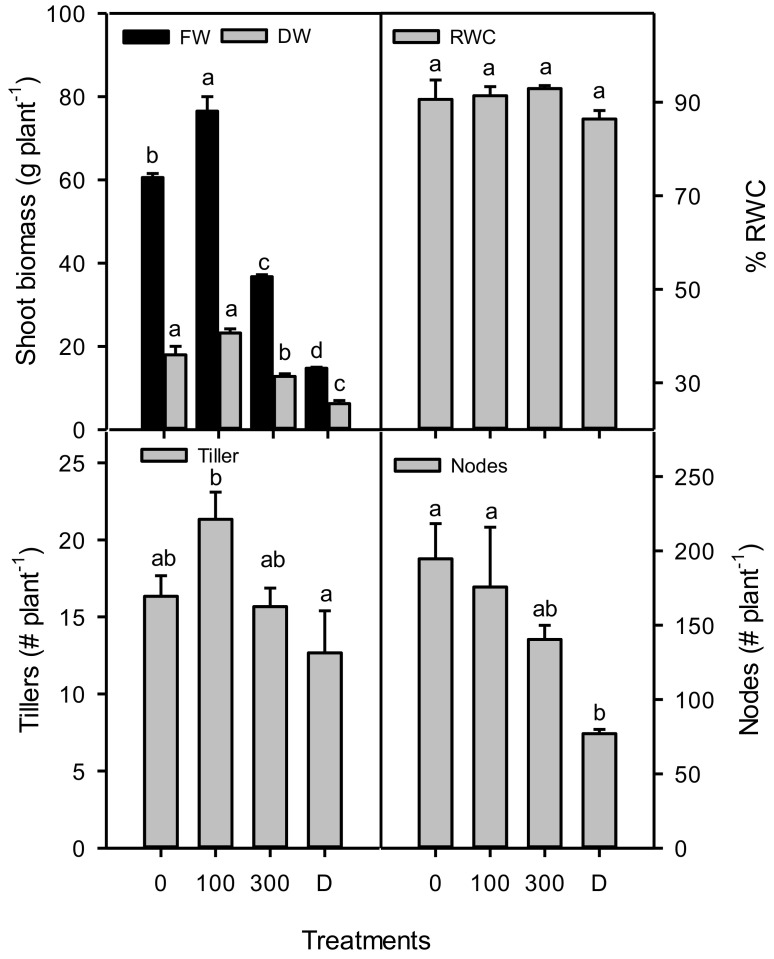
Plant fresh and dry shoot biomass, number of tillers, number of nodes of *Phragmites karka* grown at 0, 100, and 300 mM NaCl and drought. Different lower-case letters indicate significant differences due to salt treatments, according to Bonferroni’s test (*p* < 0.05).

Shoot fresh biomass was stimulated in the control at 100 mM NaCl, and it significantly decreased with an increase in the NaCl concentrations, as well as in drought treatment, while the relative water content was unchanged in all treatments (Figure 1). The number of tillers was increased only at 100 mM NaCl and decreased substantially in the other treatments compared to the control plants. The number of nodes was decreased at 300 mM NaCl and drought as compared to the other treatments. 

**Figure 2:**
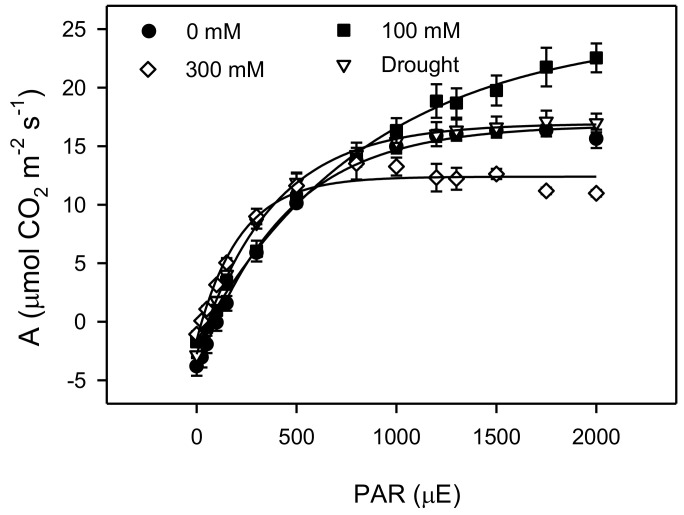
Light response curve between the net photosynthesis (A) and light intensities (PAR; 0–2500 μmol photon m^−2^ s^−1^) on leaves of *Phragmites karka* under 0, 100, and 300 mM NaCl and drought.

**Figure 3:**
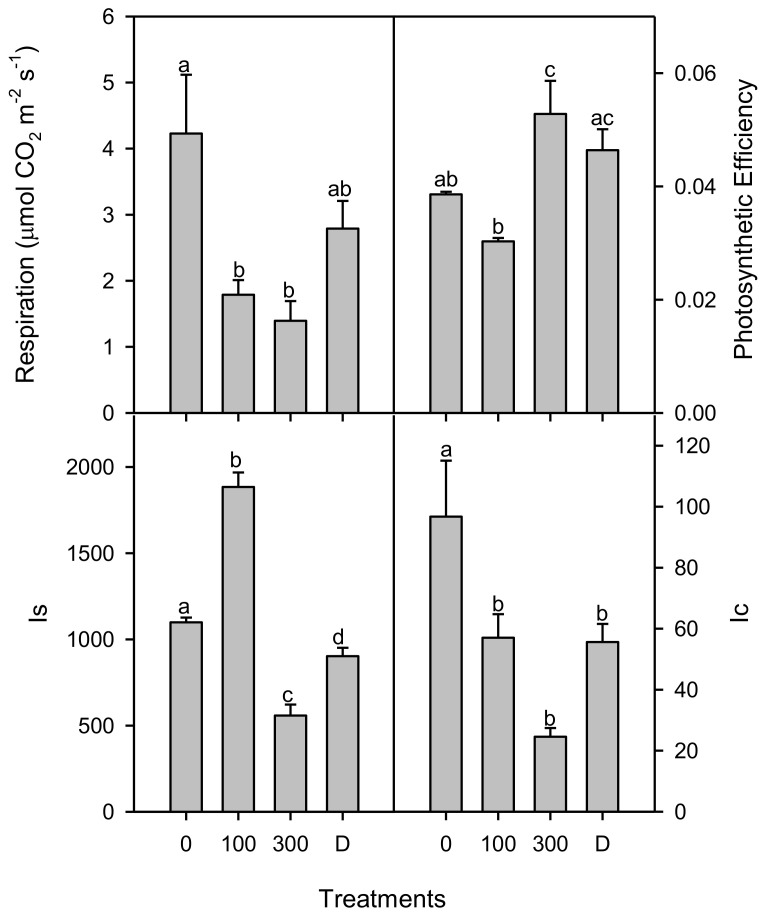
Dark respiration (Rd), compensation irradiance (Ic), saturation irradiance (Is), and photosynthetic efficiency (Φc) of *Phragmites karka* under 0, 100, and 300 mM NaCl and drought. Different lower-case letters indicate significant differences due to salt treatments, according to Bonferroni’s test (*p* < 0.05).

**Figure 4:**
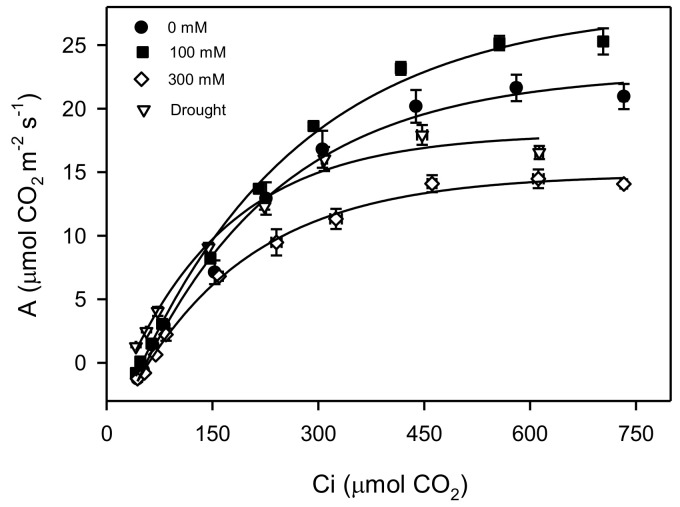
CO_2_ response curve between net photosynthesis (A) and variable intercellular CO_2_ concentrations on leaves of *Phragmites karka* under the 0, 100, and 300 mM NaCl and drought.

**Figure 5:**
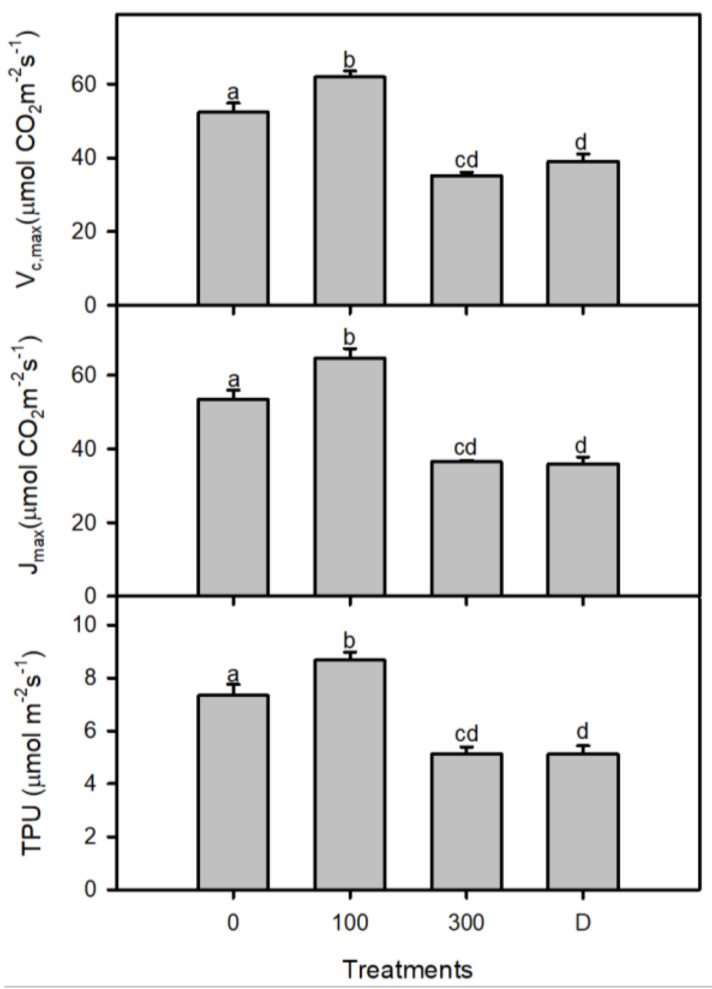
A-Ci curve was used to determine the following parameters: maximum rate of Rubisco carboxylase activity (V_c,max_), maximum rate of electron transport (J_max_), and utilization of triose phosphates (TPU) under 0, 100, and 300 mM NaCl. Different lower-case letters indicate significant differences due to salt treatments, according to Bonferroni’s test (*p* < 0.05).

An analysis of the light curves (Figure 2) showed significant changes in various treatments of salinity and drought. High salinity treatment caused an increase in the photosynthetic efficiency, but there was no significant change in the drought treatment when compared to the control plants. The saturated irradiance (I_s_) for photosynthesis was found to increase significantly (*p* < 0.001) between plants of the control and low salinity, but the I_s_ decreased significantly (about 50%) in high salt-stressed plants, with a lesser decrease (about 20%) in drought treatment with respect to control treatment. The rates of dark respiration (R_d_) and compensation irradiance (I_c_) were decreased significantly under all treatments of salinity and drought, but R_d_ was similar in the control and drought-treated plants (Figure 3) and changes in the net photosynthesis with increased CO_2_ concentration and carbon assimilation under salinity and drought conditions (Figure 4). Analyses of the *A-C_i_* curve revealed a significant improvement in the V_cmax_, J_max,_ and TPU at low salinity but decreased at 300 mM NaCl and drought treatment, as compared to the control (Figure 5). The chlorophyll fluorescence parameters were not affected under salt and drought treatments although YII and ETR were significantly increased in drought-treated plants as compared to the NaCl treatments and non-saline control plants (Table 1). 

The cellulose content was enhanced with 100 mM NaCl in *Phragmites karka*, but it was reduced substantially under drought conditions. Plants treated with the 300 mM NaCl reduced the hemicellulose content as compared to control and 100 mM NaCl. The shoot total sugar was enhanced in each stress treatments as compared to the control. Plants treated with 300 mM NaCl improved the chlorophyll (SPAD) levels as compared to the control treatments (Table 2).

**Figure 6:**
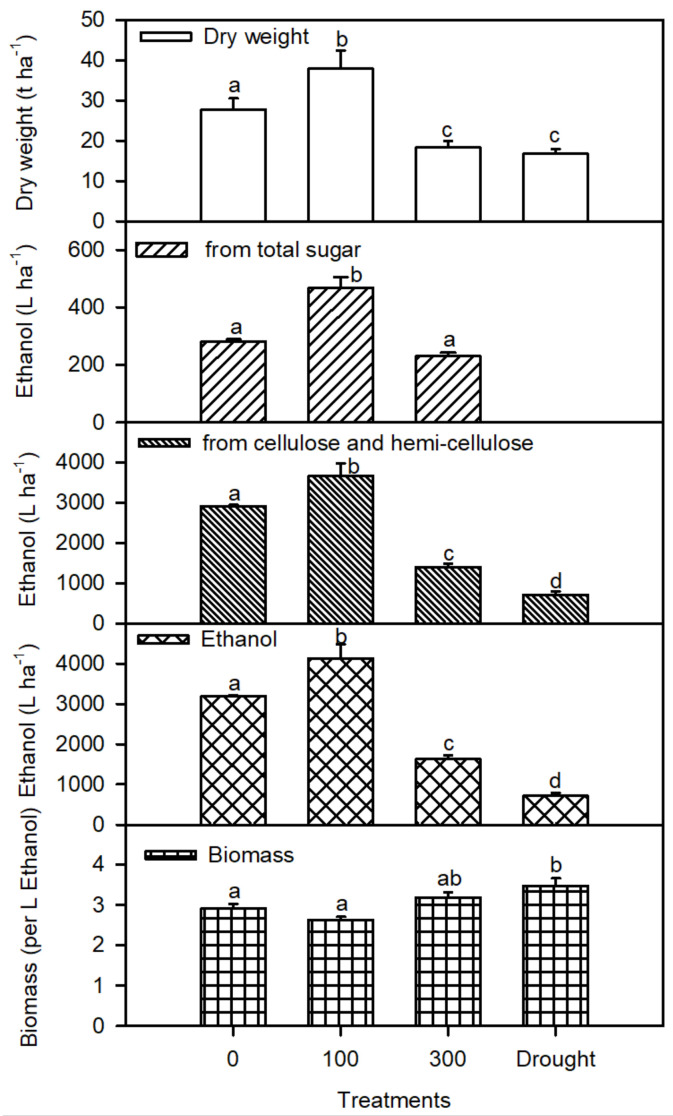
Plant dry biomass per hectare; ethanol yield per hectare from sugars; cellulose and hemicellulose; and the total ethanol yield of *Phragmites karka* under 0, 100, and 300 mM NaCl and drought. Values represent the mean ± S.E. of four replicates (*n* = 4). Different lower-case letters indicate significant differences due to salt treatments, according to Bonferroni’s test (*p* < 0.05).

The dry biomass per hectare was improved substantially at 100 mM NaCl compared to the other stress treatments. The ethanol yield was estimated from the total sugar and cellulose and hemicellulose data (Figure 6). Interestingly, the addition of 100 mM NaCl enhanced the ethanol yield per hectare by using the total sugar, cellulose, and hemicellulose in plants. The ethanol yield per hectare declined substantially under the higher salinity and drought conditions. 

## 4. Discussions

Halophyte grasses are abundantly distributed in coastal and inland saline habitats of semi-arid regions and could be a good source of lignocellulosic biomass [37]. These plants are adapted to grow under saline conditions because of their salt resistance, high water use efficiency, and fast growth rates [38]. The cultivation of these plants is highly cost-efficient, because they utilize saline water and wastelands not fit for conventional agriculture [37,38,39]. The growth of different halophyte grasses has been optimized in low and/or moderately saline conditions, such as *Phragmites australis* [40], *Phragmites communis*, *Pennisetum clandestinum* [15,41], *Panicum antidotale*, and *Spartina maritima* [42]. The optimum shoot growth of *P. karka* was observed in low salinity (100 mM NaCl), and the growth decreased in higher salinity (300 mM NaCl) and drought treatments. Our results are also in agreement with several subtropical halophyte grasses, such as *Aeluropus lagopoides*, *Sporobolus ioclados*, *Urochondra setulosa,* and *Halopyrum mucronatum* that showed the optimum growth under non-saline conditions [43]. Besides *P. karka*, other species belonging to the genus *Phragmites* showed dose-dependent growth responses under saline conditions [40,41]. Therefore, we could suggest that our test species is one of the best candidates for using the sustainable utilization of saline land, particularly in arid and semi-arid regions of the world. 

The rate of the leaf gas exchange varies with the duration and levels of salinity and drought conditions [44]. The ability of a plant to maintain its chlorophyll level, stomatal conductance, and rate of efficient CO_2_ assimilation under saline conditions are closely related to the salt tolerance ability of the plant [45]. The photosynthetic efficiency of *P. karka* was decreased with an increase in the salinity; however, it remained comparable in drought treatment with the non-saline control (0 mM NaCl). 

The survival of plants under drought and salinity without compromising the biomass is difficult; however, the salt-resistant plant maintains an optimum water use efficiency and rate of photosynthesis and fast growth rate. Under high salinity, plants improve their water use efficiency by decreasing their transpiration rate [46]; however, this reduces the CO_2_ uptake, and therefore, photosynthesis is inhibited. *Phragmites karka* optimized net photosynthesis with a minimum water loss and favored higher photosynthetic rates (A) at 100 mM NaCl. However, at a higher salinity and under drought treatment, plants ensured their survival but with a growth reduction. A similar strategic reduction in photosynthetic efficiency and growth was reported for many halophytes under various abiotic stresses, such as *Desmostachya bipinnata* [47], *Aeluropus lagopoides*, and *Sporobolus tremulus* [48], under various abiotic stresses [45,47]. An effective CO_2_ and water exchange is necessary for the survival of plants under stress conditions [47,48]. *Phragmites karka* exhibited higher energy requirements with increasing concentrations of NaCl, as indicated by an increase in compensation irradiance (Ic). High salt concentrations (300 mM NaCl) caused a reduction in photosynthetic machinery, which leads to a decrease in the level of Is. However, unutilized light by the photosystem may trigger photochemical damage [49]. Our data is in agreement with several published reports [44,49,50]. 

The data extracted from A-Ci curves showed a significant decrease in the maximum rate of Rubisco carboxylase activity (Vc, max), maximum rate of photosynthetic electron transport to regenerate Ribulose-1,5-bisphosphate (Jmax), and utilization of triose phosphate (TPU) under higher salinity and drought conditions. The reduction of TPU in *P. karka* indicates that the synthesis of sucrose/starch might be inhibited due to the reduced regeneration of phosphate (Pi) under stress conditions [15]. In addition, it may also cause growth inhibition under stress conditions, which is also evident in the lower values of the cellulose and hemicellulose contents in *P. karka* plants growing at 300 mM NaCl and drought [51,52]. Any alteration in the electron transport (ETR) disturbs the availability of the electron acceptors (like NADP+) and utilization of ADP that ultimately limits the regeneration of ribulose-1,5-bisphosphate [52]. Hence, it can be suggested that, under high salinity and drought conditions, the biochemical efficiency of the photosynthetic apparatus in *P. karka* plants decreased due to the colimitation of Vc, max, Jmax, and TPU.

The chlorophyll fluorescence data provides detailed insights into the integrity and efficient functioning of photosystem II (PSII). The maximum quantum yield of photosystem II (Fv/Fm) indicates the level of photoinhibition [53]. In the present study, unaffected Fv/Fm in all treatments suggested that there was no sign of photoinhibition, and it indicated the resilient ability of *P. karaka* in response to salt and drought stress. Our findings are also in agreement with other salt-resistant plants such as *Urochondra setulosa* and other halophytes [54,55]. It is also supported by the higher values verified for the maximum electron transport rates (ETR) in this experiment, where higher electron transport rates (ETR) were found in all treatments, especially under drought conditions. Non-Photochemical Quenching (NPQ) is an indicator of dissipating nonradiative energy from the light-harvesting complex (LHC II) of PSII that prevents the overreduction of ETC and therefore avoids damage to the photosynthetic process. Growth inhibition under water stress is caused by lower leaf expansion (due to less turgid cells, *P. karka* buffered the loss of the photosynthetic active leaf surface area by maintaining a high electron transport rate and Φ PSII under drought [3]). A higher NPQ was observed in *P. karka* at a higher salinity, indicating the efficient heat dissipation mechanism under a saline condition so NPQ serves as an index of stress for the plant [56,57]. Under severe stress situations, *P. karka* used a regulated and effective Y (NPQ) in this study to release absorbed light energy as heat that ultimately caused no change in the nonregulated process Y(NO). A similar strategy of heat dissipation has been documented in *Paspalum paspalodes* and *Paspalidium geminatum* [48]. The upregulation of the xanthophyll cycle and synthesis of photoprotective compounds such as carotenoids and the activity of photorespiration also support plant heat dissipation, which is critical to avoiding photosystem II damage under suboptimum situations [48,58,59].

The cell wall composition in grasses mostly consists of cellulose microfibrils interlinked with glucuronoarabinoxylans and polyphenolic depositions [60]. The synthesis of higher cellulose and hemicellulose in *P. karka* under saline conditions protects and supports the plant from lodging and higher light gaining, which promote growth and seedling vigor under saline and drought stress [61]. Generally, plants can reduce the cellulose synthesis and influence lignin accumulation under stress [62,63]. However, in salt-tolerant plants, the crude fiber, cellulose, and hemicellulose contents increased under salt stress [63]. Higher cellulose, hemicellulose, and total sugars in *P. karka* at a low salinity (100 mM NaCl) treatment suggest it could be a source of lignocellulose for bioethanol production in salt-affected lands. The cellulosic and hemicellulosic contents of *P. karka* are also comparable with the other bioenergy crops, such as *Cynodon dactylon* (35.7% cellulose, 25% hemicellulose) and *Panicum virgatum* (16.8% cellulose, 27.8% hemicellulose) [34].

Plants have been known as promising energy feedstock for ages and used for bioenergy production due to their lower cultivation cost, lower carbon dioxide emissions, and it is abundant in nature [64,65]. The per hectare dry biomass of *P. karka* was improved substantially at 100 mM NaCl. Higher per hectare aboveground dry biomass is reported in different feedstock crops for bioethanol [64], such as sweet sorghum [65]. The ethanol yield from the total sugars, cellulose, and hemicellulose contents were also estimated for *P. karka* in this study. Interestingly, the addition of 100 mM NaCl in the growth medium enhanced the yield of the ethanol per hectare by using the total sugar, cellulose, and hemicellulose in plants. Hence, the prospect of *P. karka* as feedstock for ethanol is probably very high, which could be helpful in utilizing the saline wastelands, as well as minimizing the energy crises and land competition for food and fuels. 

## 5. Conclusions

This study reflects the contributions of different photochemical, stomatal, and biochemical factors on the growth performance, dry biomass, and predicted bioethanol production of *Phragmites karka* under dry, arid saline conditions. This study shows that the higher saturated irradiance (Is) of light, maximum rate of Rubisco carboxylase activity (Vc, max), maximum rate of electron transport (Jmax), and utilization of triose phosphates (TPU) are responsible for the change in the growth of *P. karka* under suboptimum conditions. An increase in the respiratory rates exerts positive effects on the plant performance and metabolism by providing more energy to invest in the biomass and ethanol production. Growth inhibition under higher salinity and drought could be attributed to limited stomatal closure and decreased CO_2_ assimilation. *P. karka* can be grown and produce a higher dry biomass and ethanol yield per hectare in saline and arid areas and could therefore be used as a sustainable biofuel crop. An increase in the maximum quantum yield, effective quantum yield, and lower photochemical quenching parameters are important in protecting plants by dissipating excessive energy, especially in drought conditions. These results clearly postulate that *P. karka* can be cultivated in areas of low salinity with the optimal photosynthetic performance. The production of higher ethanol and lignocellulosic contents in salinity can be useful in reducing the energy crises, land competition, and environmental protection. 

## Figures and Tables

**Table 1:** Chlorophyll fluorescence parameters (Fv/Fm, maximum photochemical quantum yield of PSII; Y(II), effective photochemical quantum yield of PSII; coefficient of photochemical quenching (qP); Non-photochemical quenching (NPQ) Y(NPQ), yield for heat dissipation; Y(NO), and yield of non-photochemical; and ETR, electron transport rate under saline and drought conditions. Different lower-case letters indicate significant differences due to salt treatments, according to Bonferroni’s test (*p* < 0.05).

Treatments	Fv/Fm	Y(II)	qP	NPQ	Y(NO)	Y(NPQ)	ETR
0	0.81 ± 0.005a	0.51 ± 0.012a	0.70 ± 0.015abc	0.59 ± 0.026b	0.30 ± 0.006a	0.18 ± 0.008abc	40.77 ± 0.91a
100	0.82 ± 0.006a	0.50 ± 0.020a	0.68 ± 0.033b	0.66 ± 0.093b	0.29 ± 0.018a	0.19 ± 0.020b	40.22 ± 1.63a
300	0.82 ± 0.005a	0.51 ± 0.030a	0.68 ± 0.032b	0.63 ± 0.057b	0.29 ± 0.013a	0.18 ± 0.020b	41.14 ± 2.45a
Drought	0.81 ± 0.004a	0.59 ± 0.014b	0.78 ± 0.017ac	0.47 ± 0.092a	0.27 ± 0.015a	0.13 ± 0.019ac	47.12 ± 1.12b

**Table 2:** Shoot cellulose (%), hemicellulose (%), total sugar (mg/g DW), and leaf chlorophyll (SPAD arbitrary values) of *Phragmites karka* under 0, 100, and 300 mM NaCl and drought. Values represent the mean ± S.E. of three replicates (*n* = 4). Different lower-case letters indicate significant differences due to salt treatments, according to Bonferroni’s test (*p* < 0.05).

Treatments	Cellulose	Hemicellulose	Soluble Sugar	Chlorophyll
Control	29.17 ± 1.14b	22.31 ± 1.11b	51.91 ± 4.21a	40.51 ± 0.56a
100 mM NaCl	34.56 ± 1.20c	20.77 ± 2.00a	78.61 ± 2.73c	43.91 ± 0.36a
300 mM NaCl	26.67 ± 1.49b	17.72 ± 1.56a	79.40 ± 2.97c	47.47 ± 0.45b
Drought	20.06 ± 0.63a	14.82 ± 0.44a	69.50 ± 8.27b	39.69 ± 1.99a

## Data Availability

Not applicable.
